# Supplementary figures and images for: Gasdermin D-mediated neutrophil pyroptosis drives inflammation in psoriasis
Source: eLife. 2024 Dec 24;13:RP101248. doi: 10.7554/eLife.101248 (PMC11668524; doi:10.7554/eLife.101248)

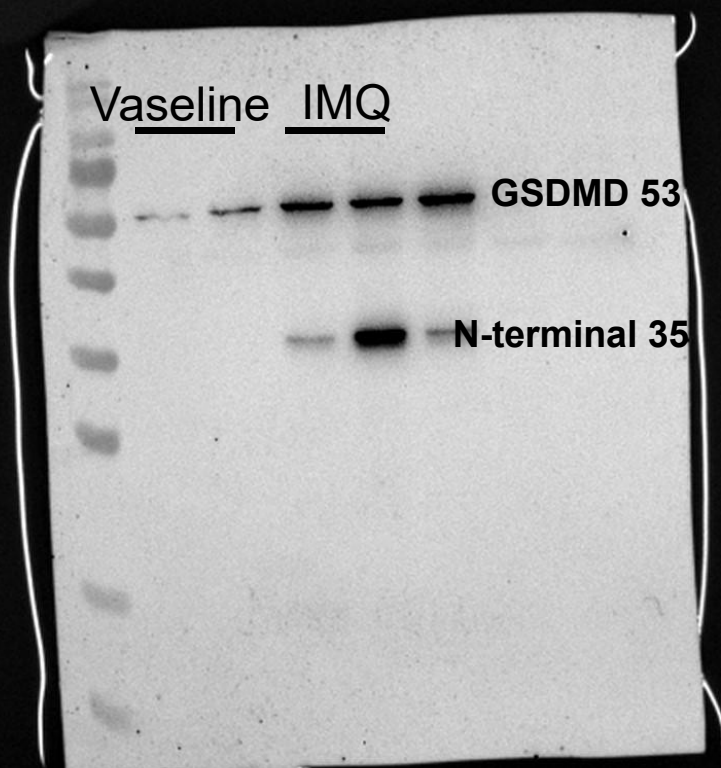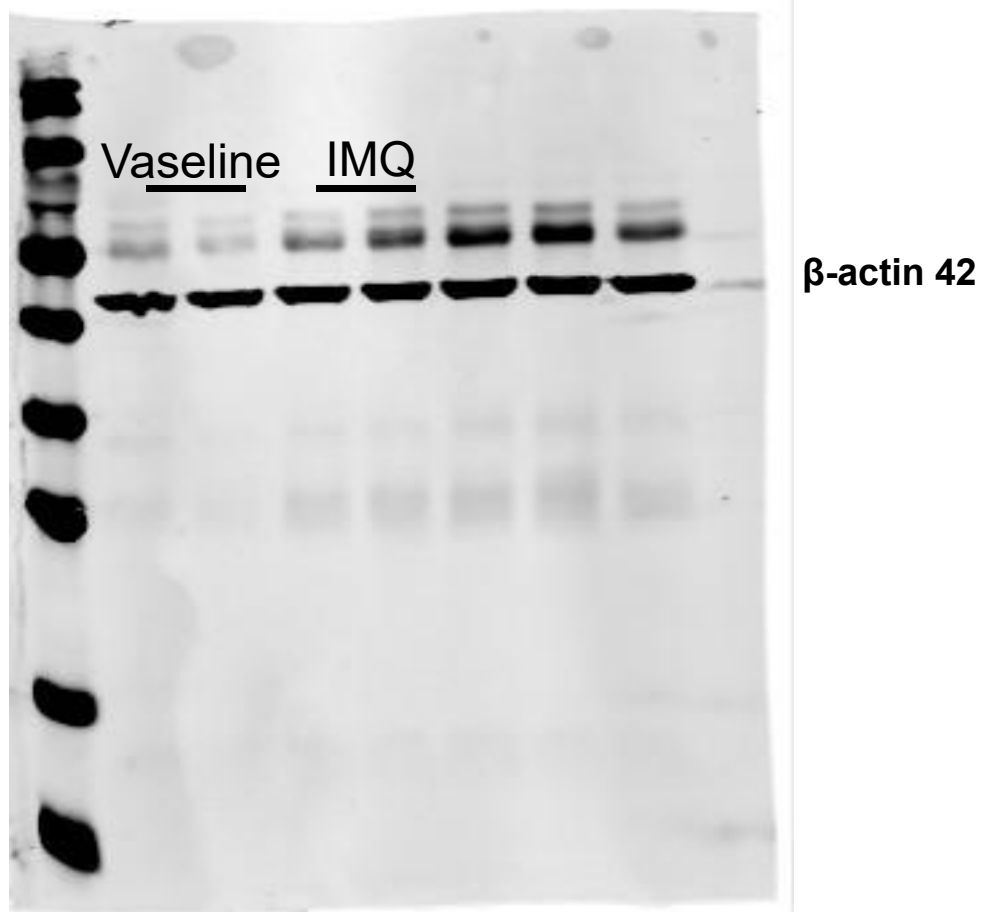

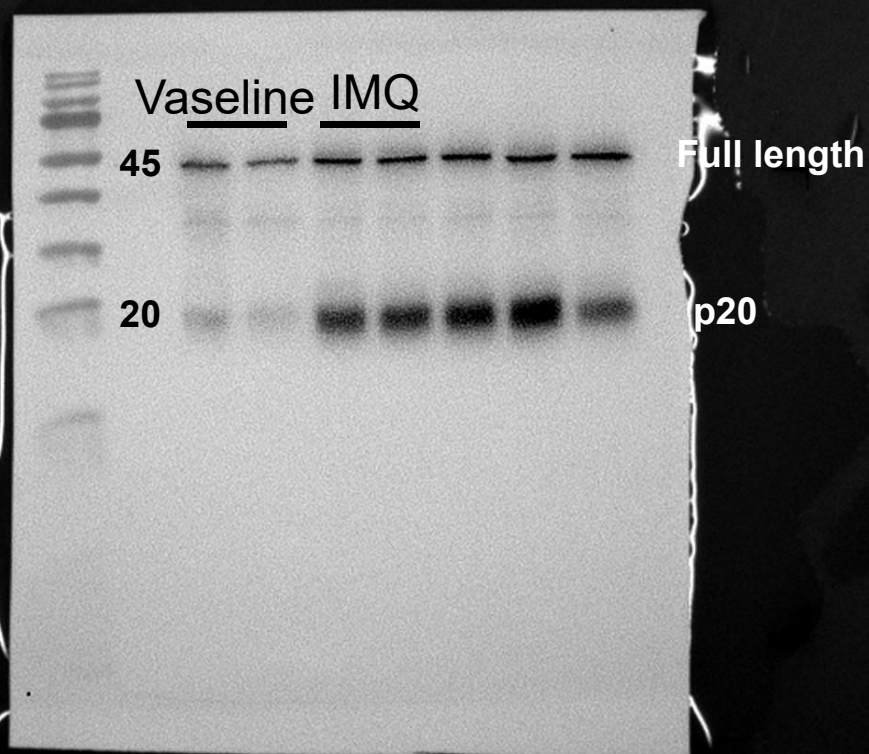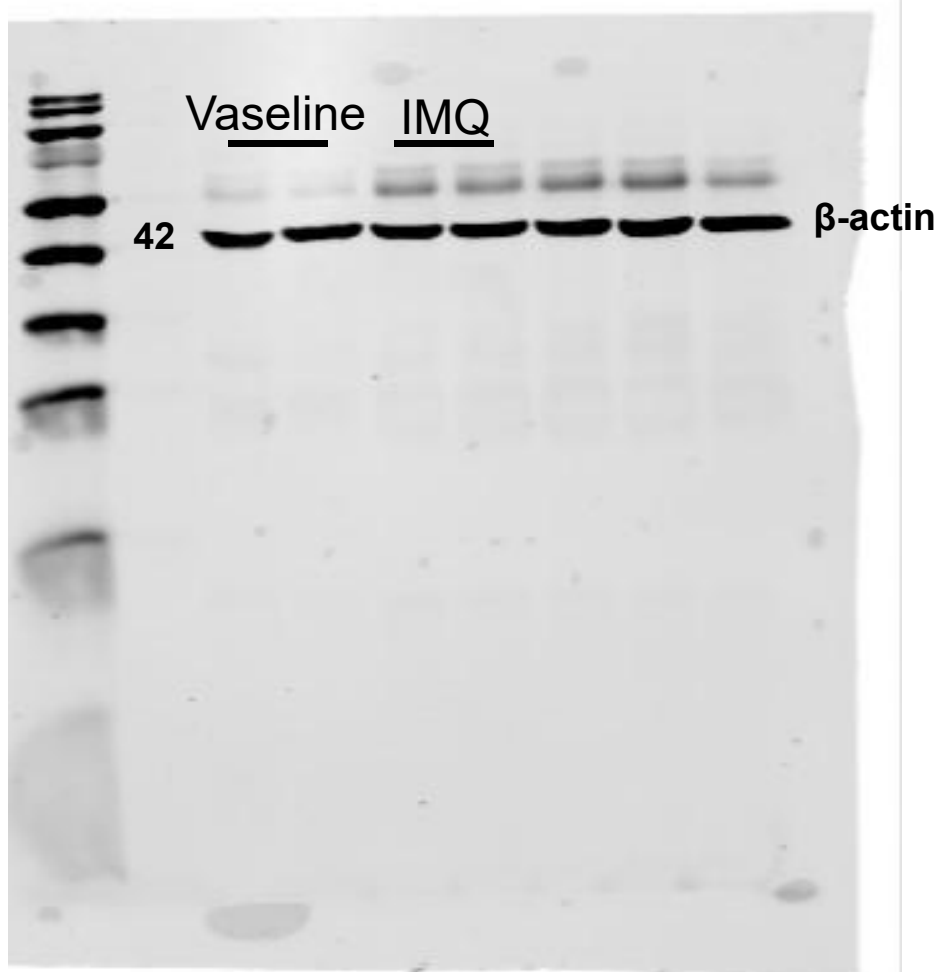

Supplement: Figure 1—source data 1. [file elife-101248-fig1-data1.pdf]

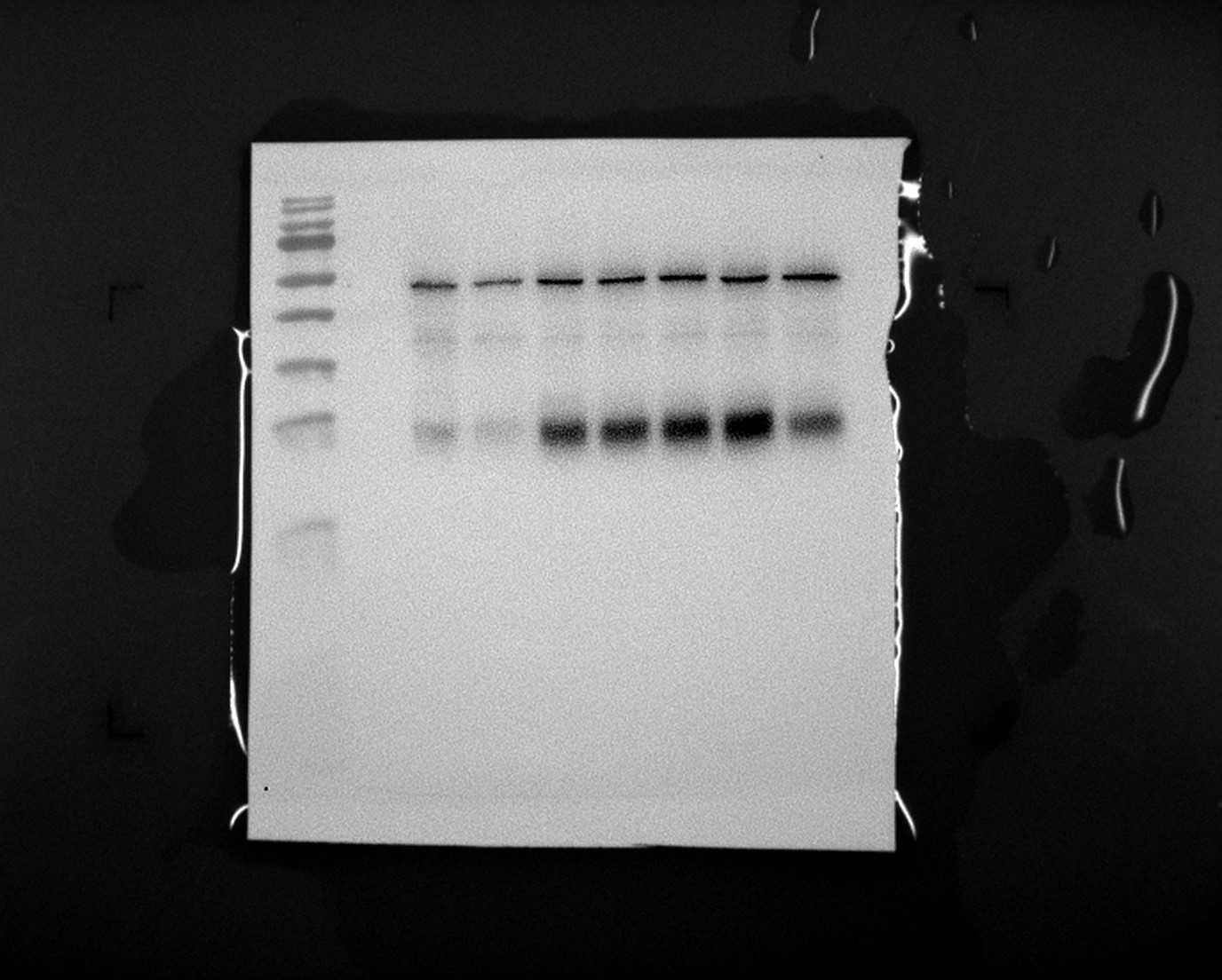

Supplement: Figure 1—source data 2. [file elife-101248-fig1-data2.zip › Figure1-source data2/casp1.jpeg]

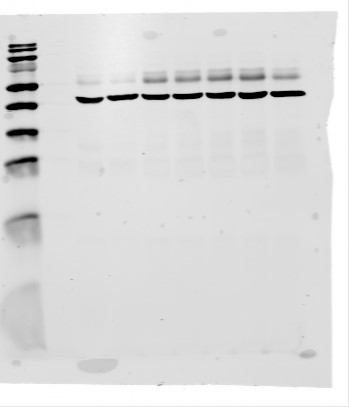

Supplement: Figure 1—source data 2. [file elife-101248-fig1-data2.zip › Figure1-source data2/caspase1-actin.jpeg]

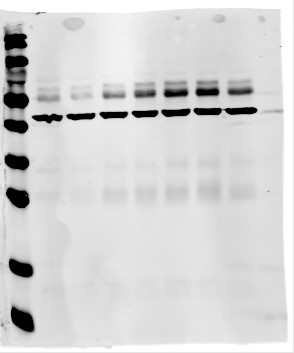

Supplement: Figure 1—source data 2. [file elife-101248-fig1-data2.zip › Figure1-source data2/gsdmd-actin.jpeg]

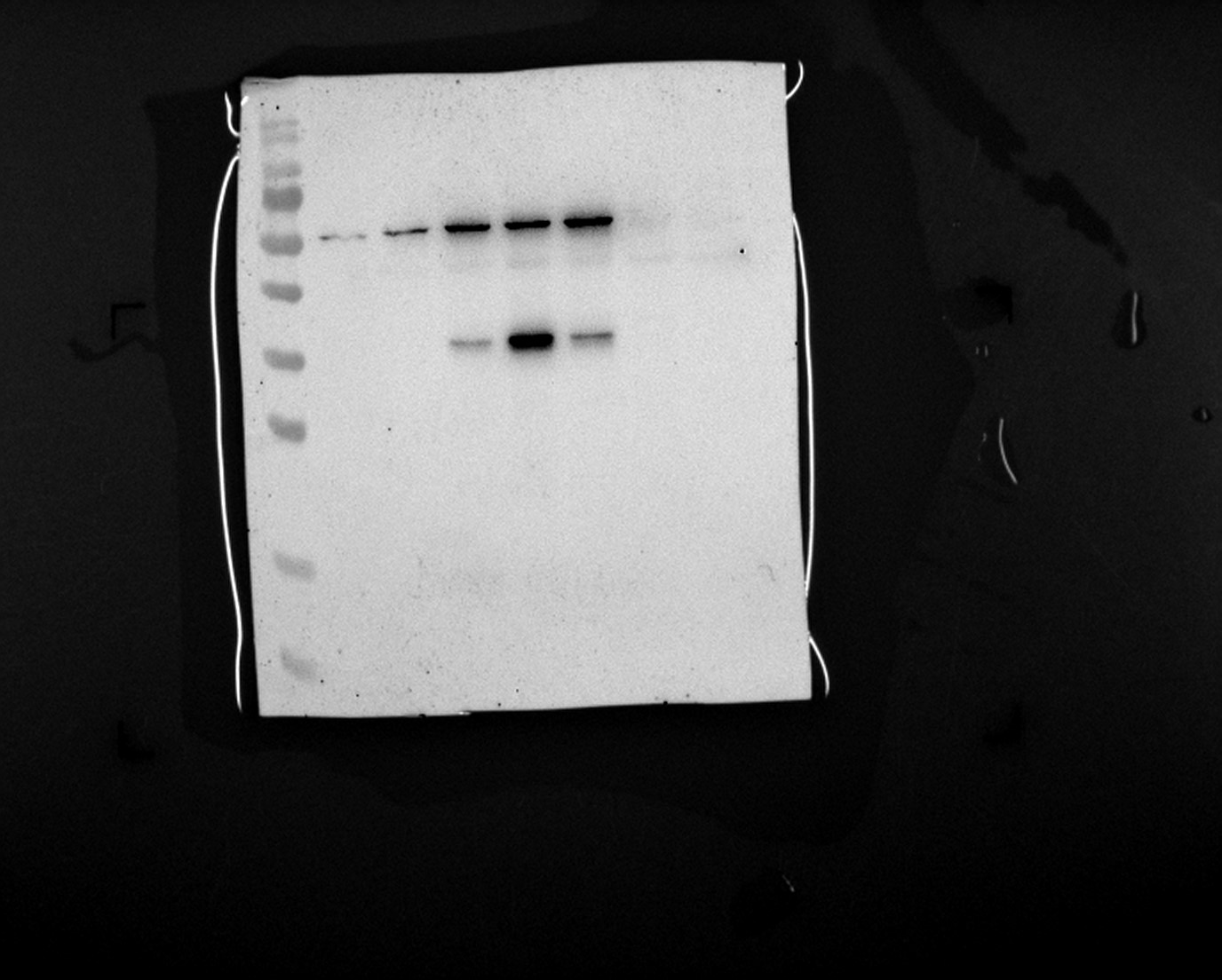

Supplement: Figure 1—source data 2. [file elife-101248-fig1-data2.zip › Figure1-source data2/gsdmd.jpeg]

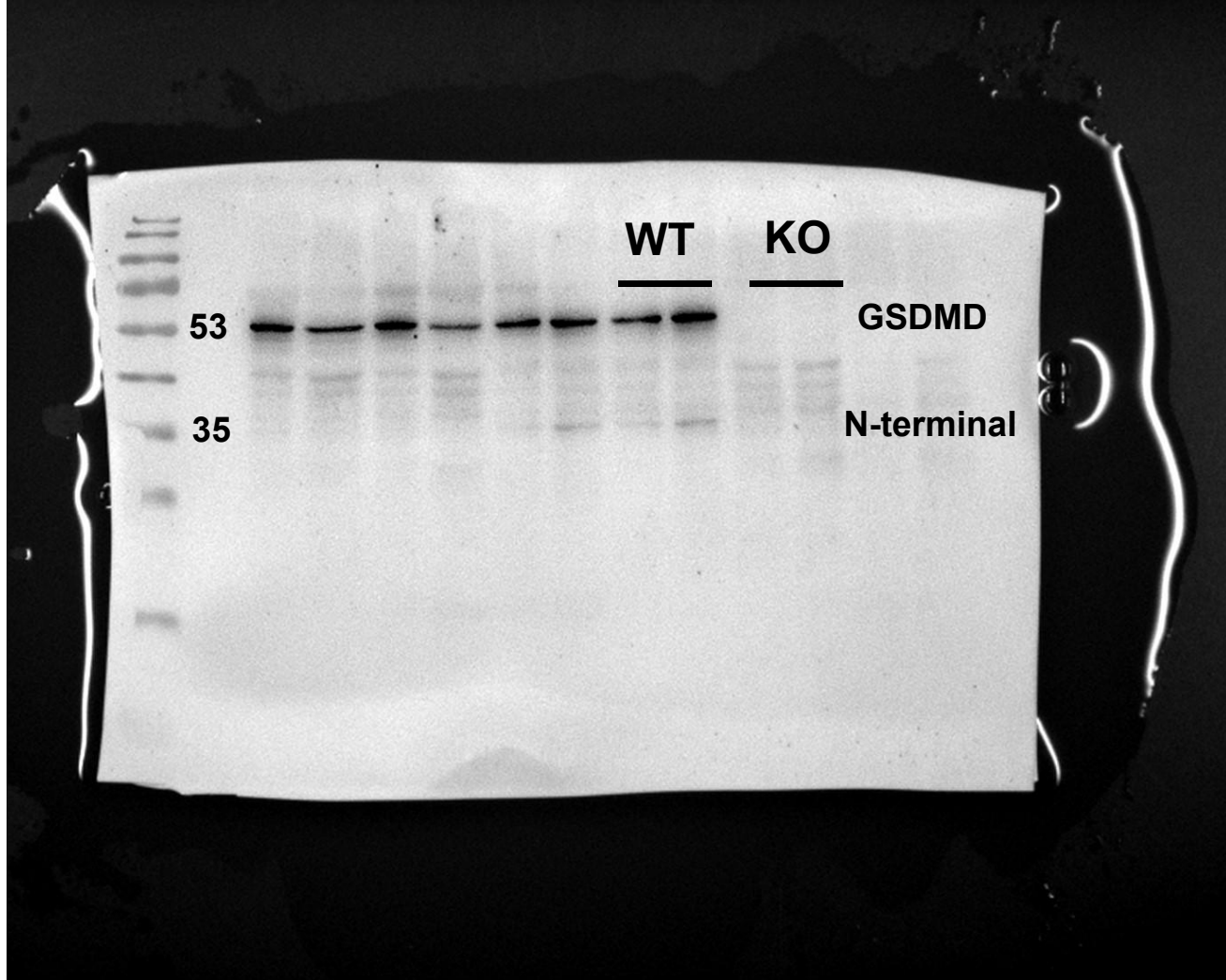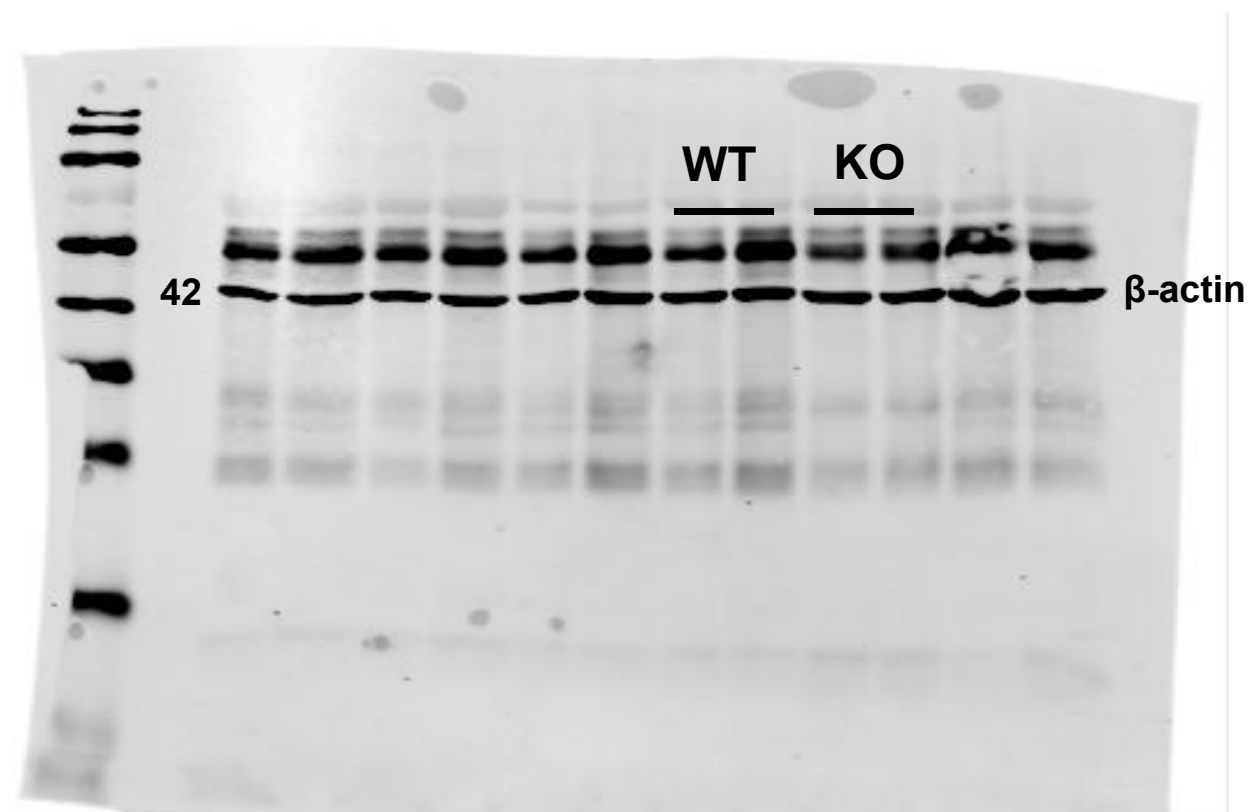

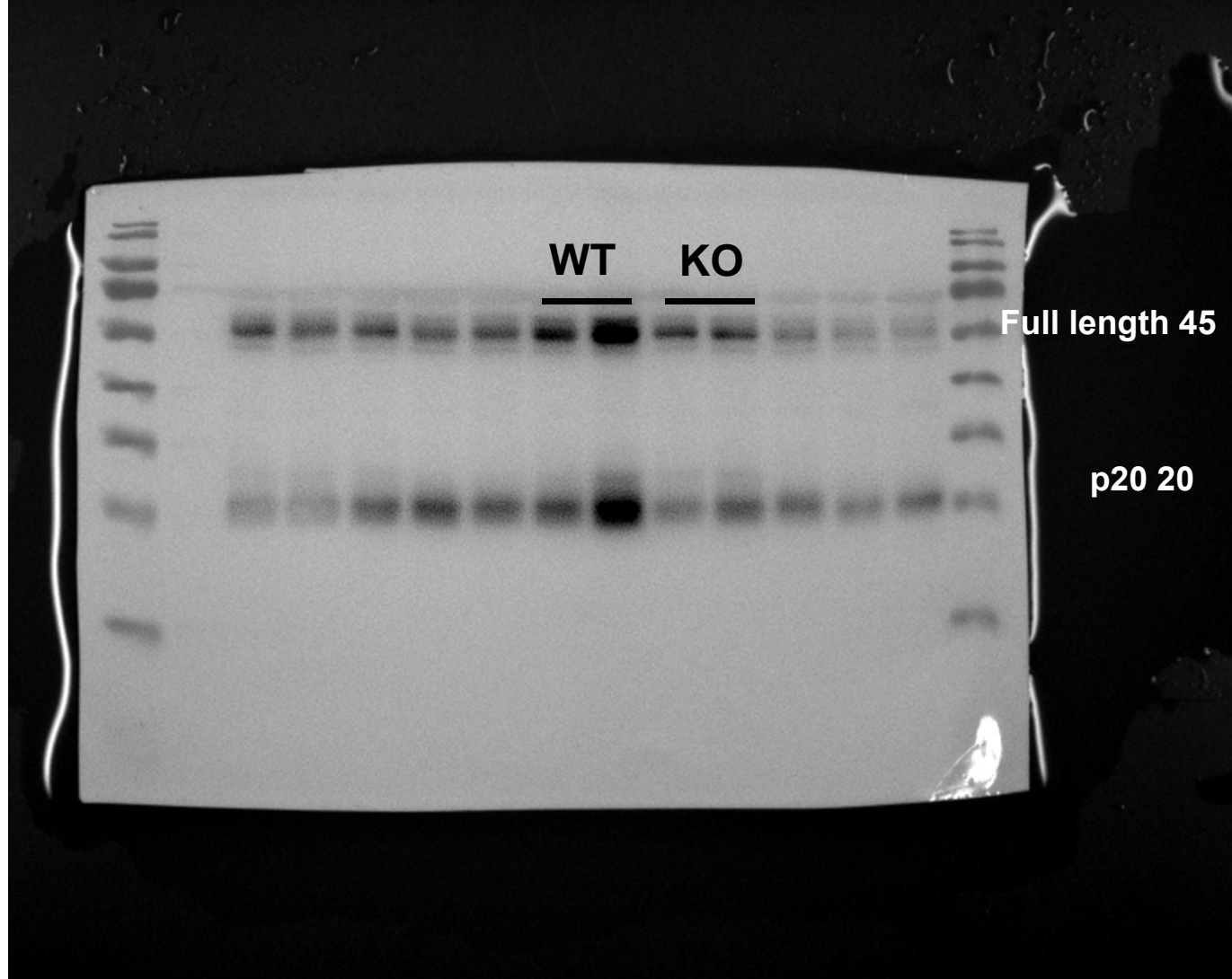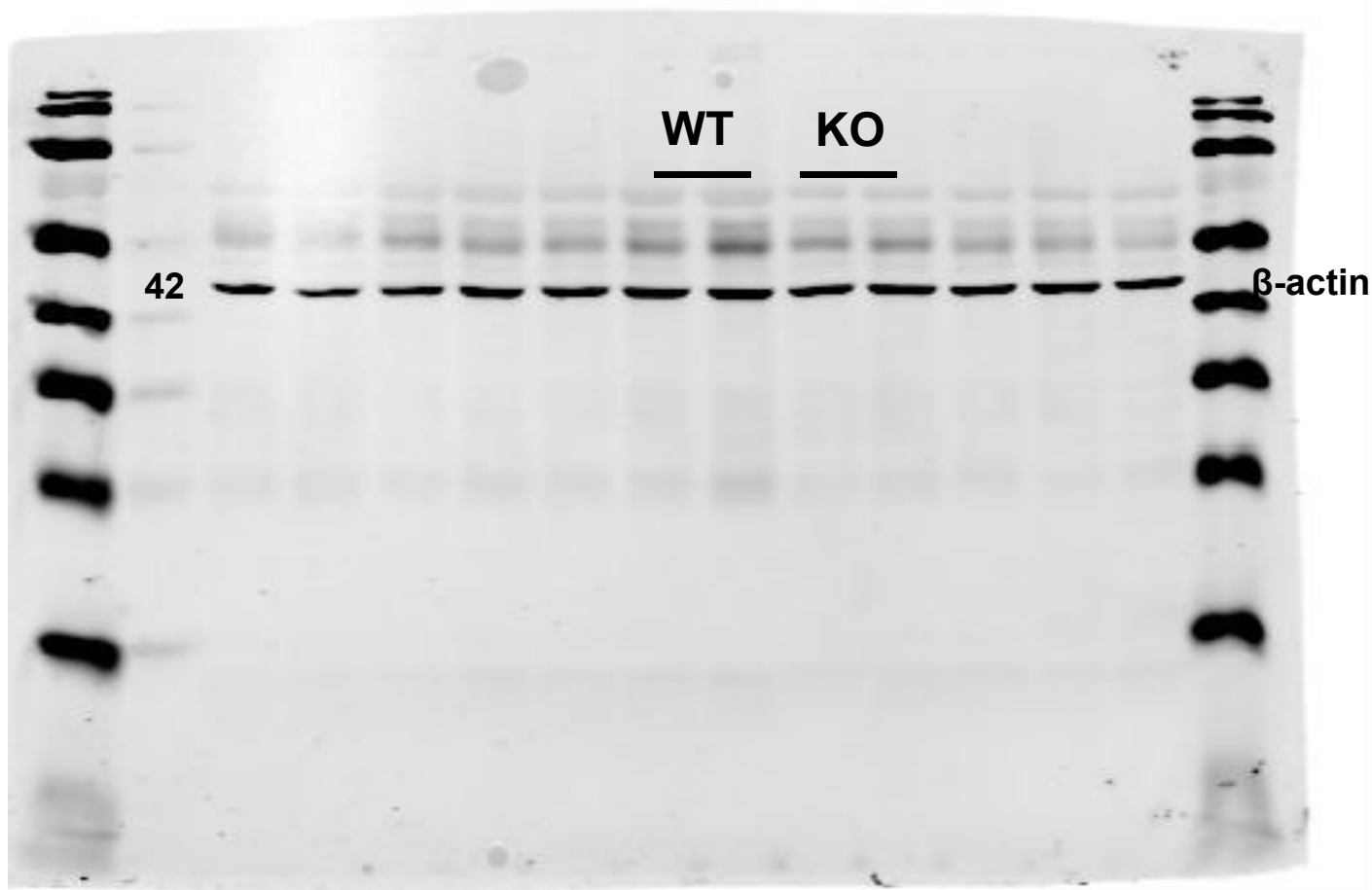

Supplement: Figure 2—source data 1. [file elife-101248-fig2-data1.pdf]

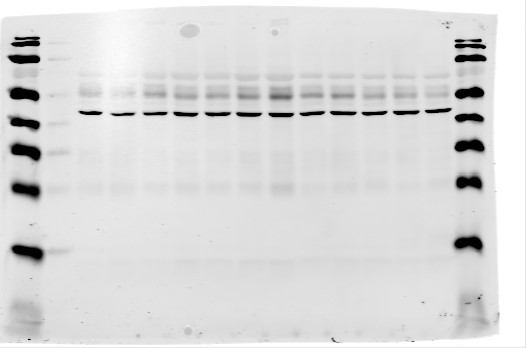

Supplement: Figure 2—source data 2. [file elife-101248-fig2-data2.zip › Figure2-source data2/casp1-actin.jpeg]

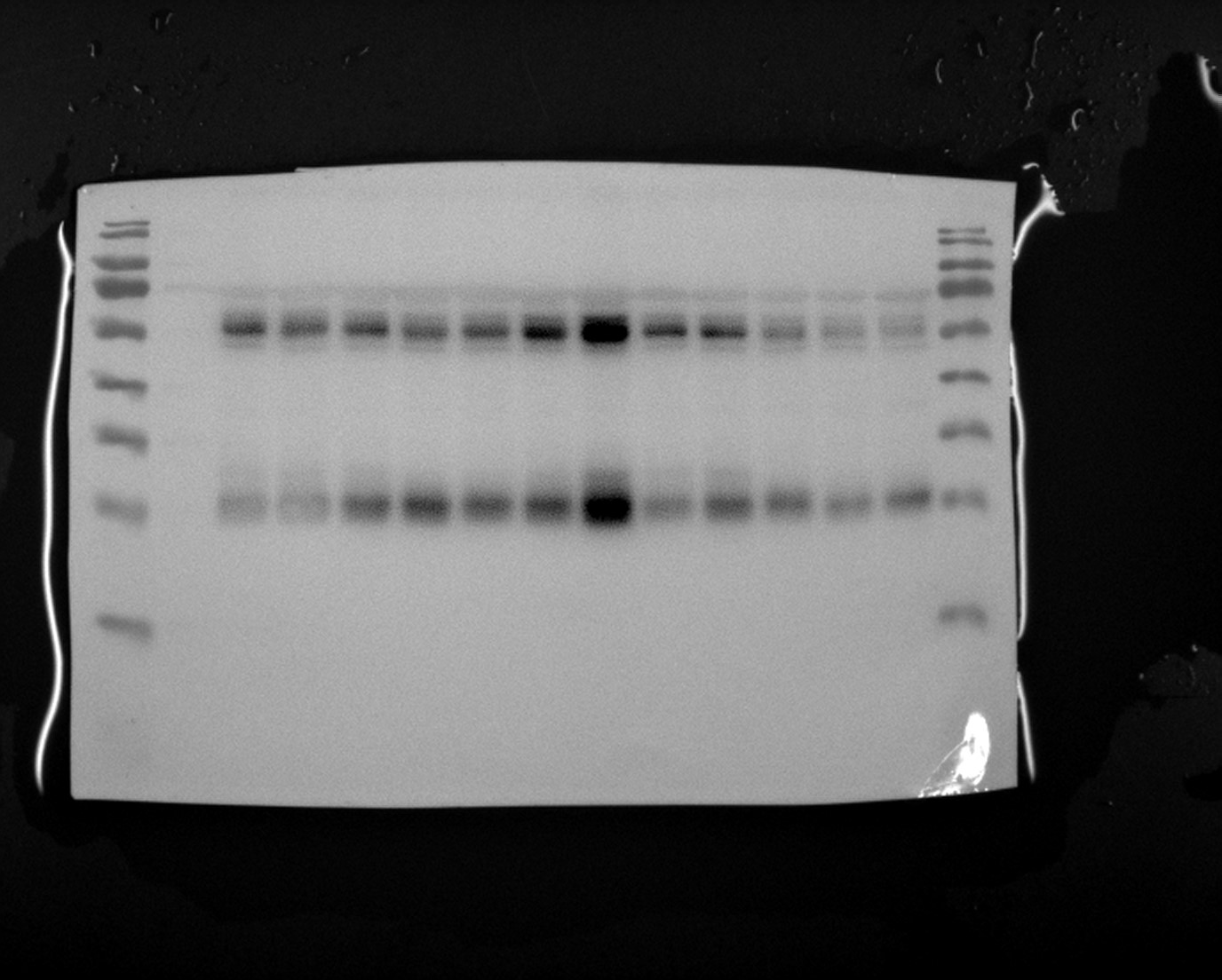

Supplement: Figure 2—source data 2. [file elife-101248-fig2-data2.zip › Figure2-source data2/casp1.jpeg]

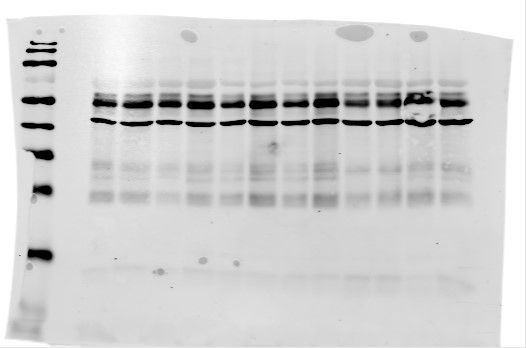

Supplement: Figure 2—source data 2. [file elife-101248-fig2-data2.zip › Figure2-source data2/gsdmd-actin.jpeg]

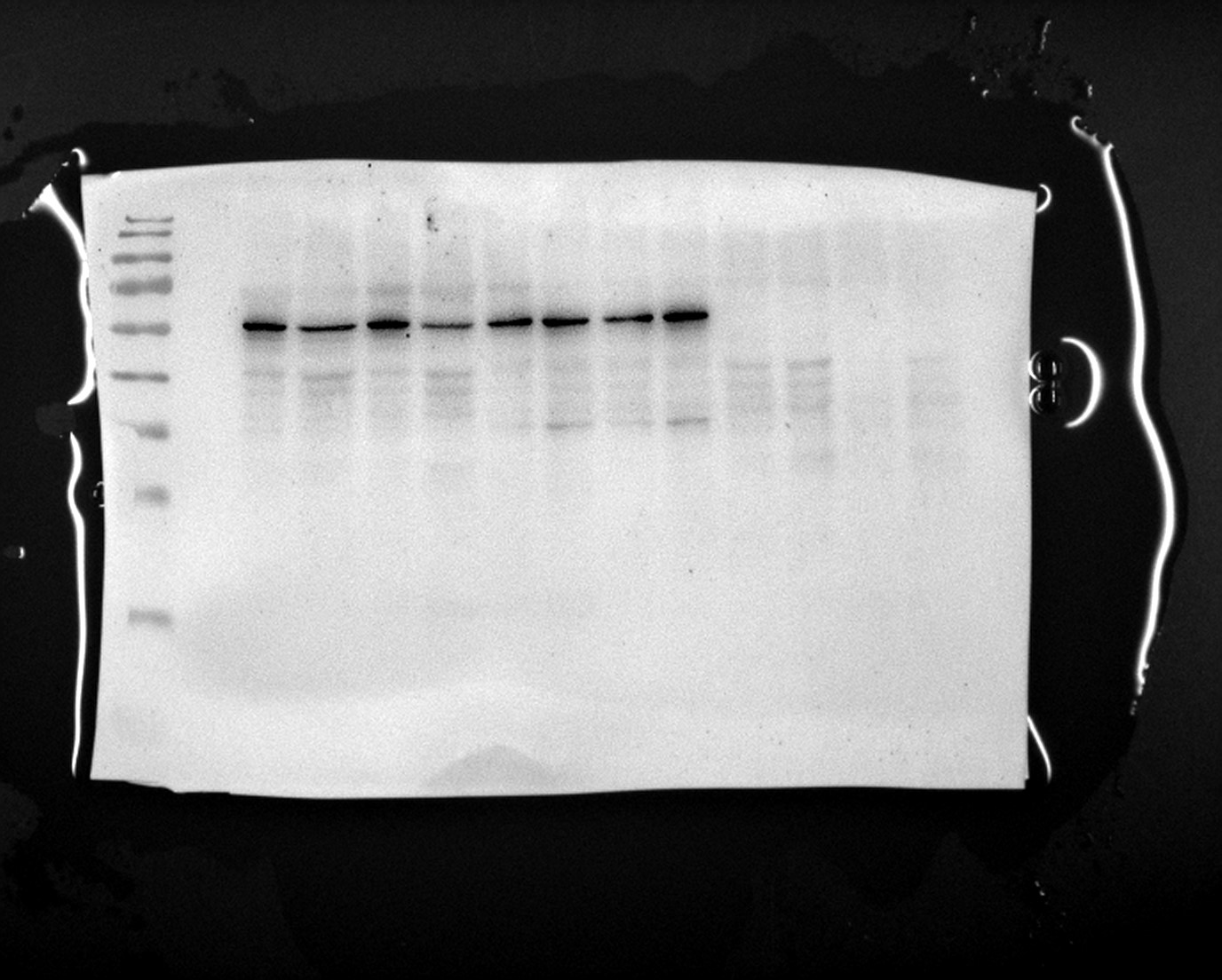

Supplement: Figure 2—source data 2. [file elife-101248-fig2-data2.zip › Figure2-source data2/gsdmd.jpeg]

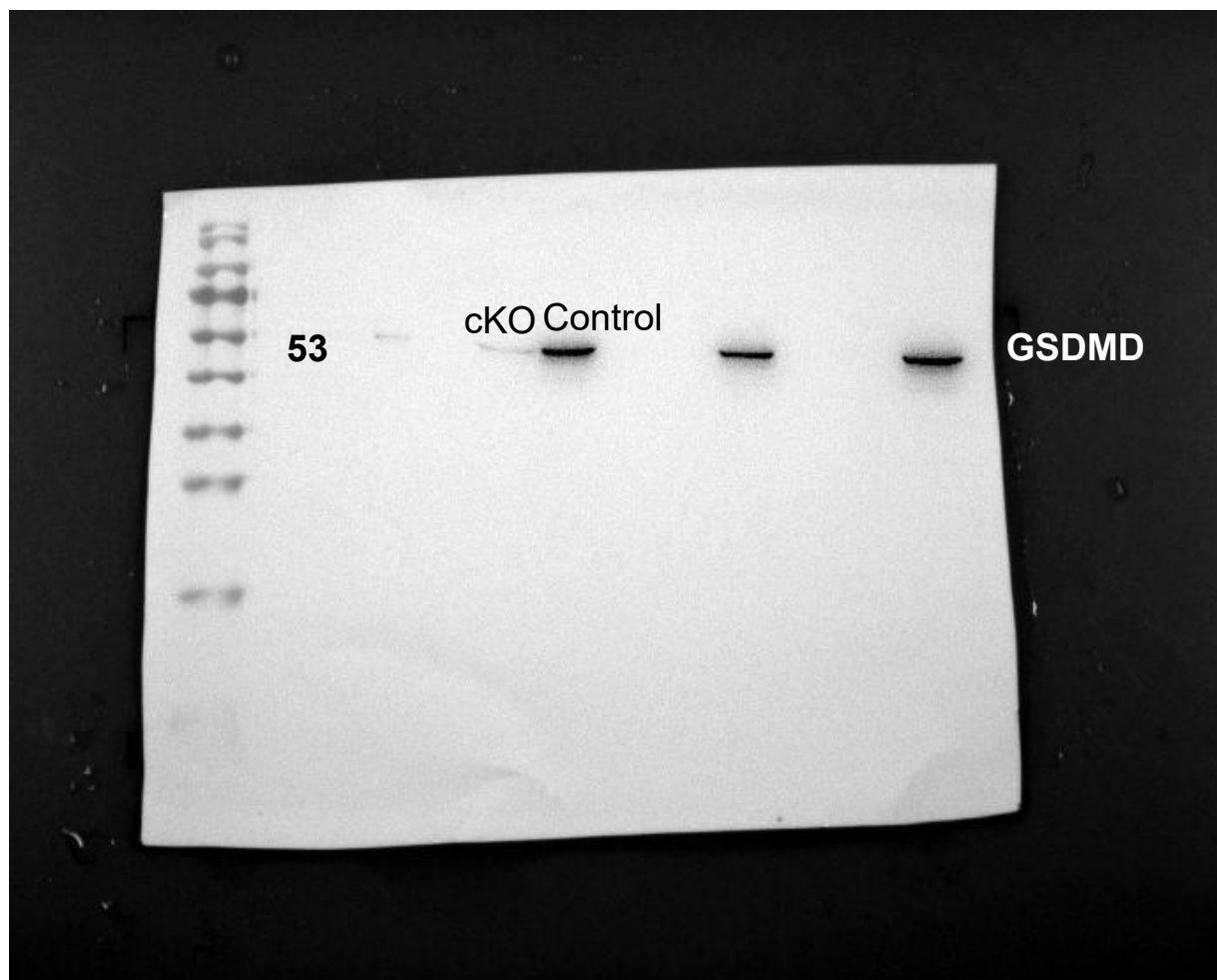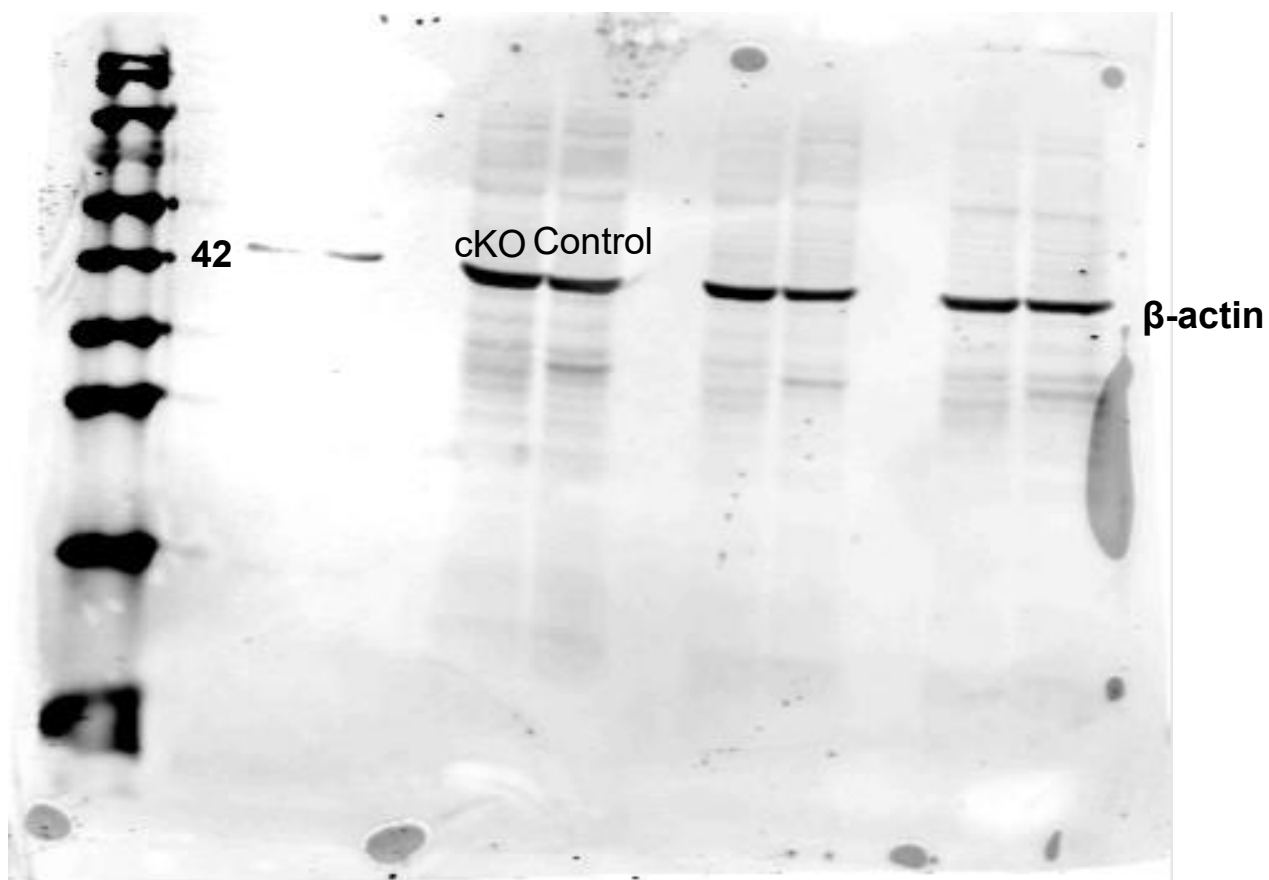

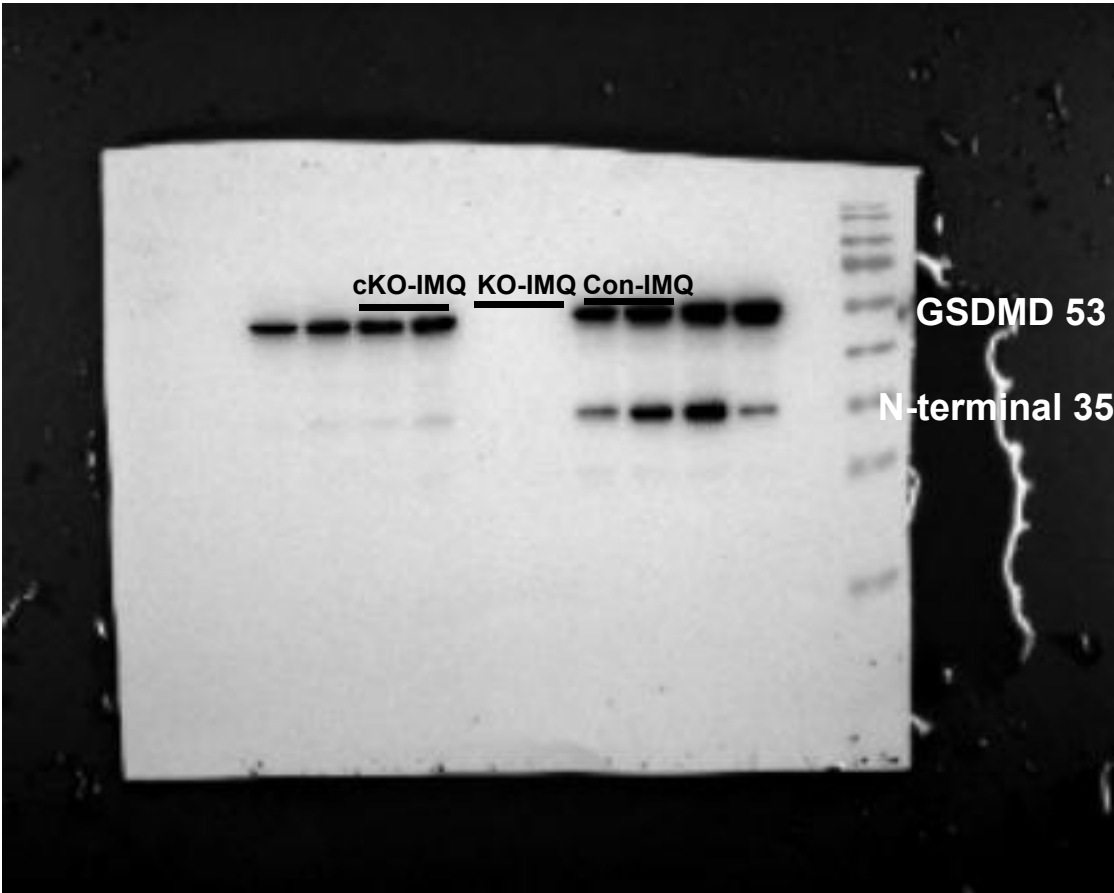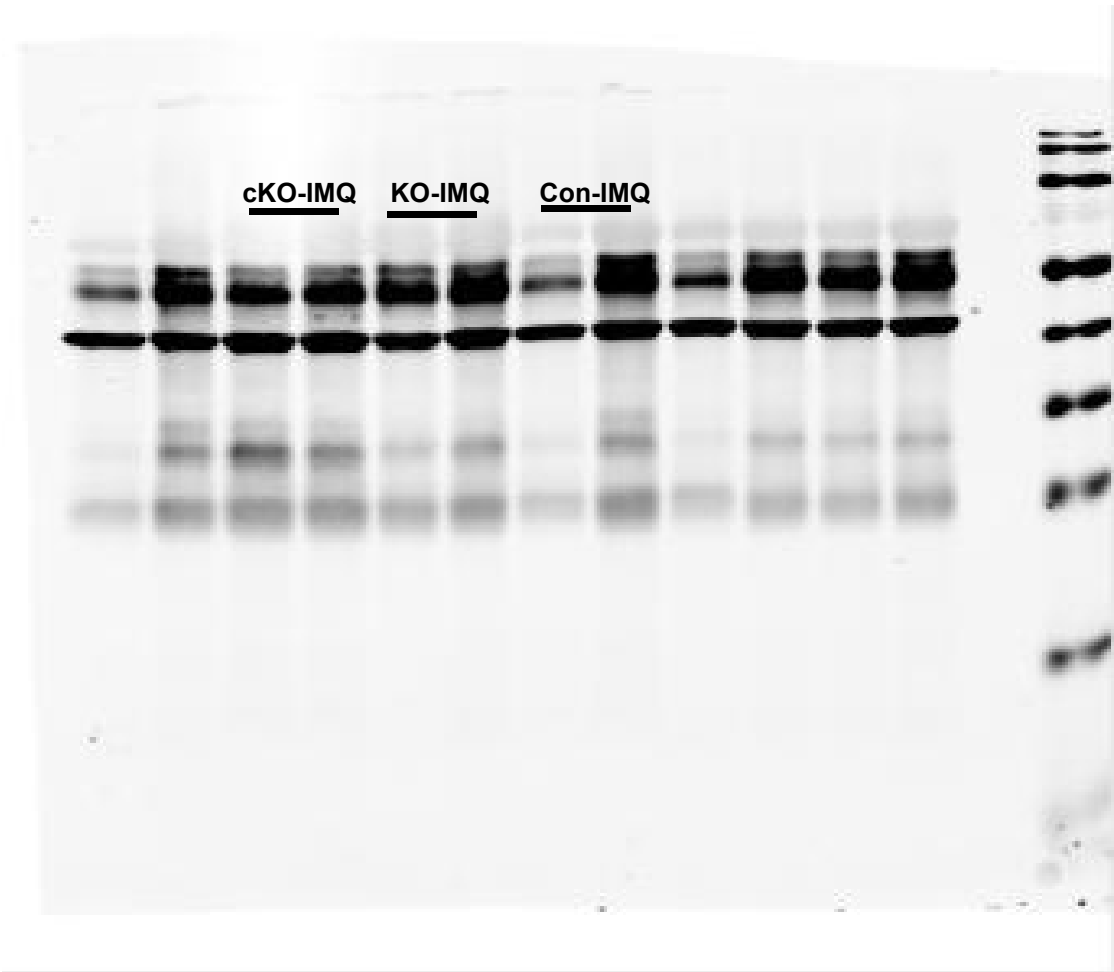

Supplement: Figure 4—source data 1. [file elife-101248-fig4-data1.pdf]

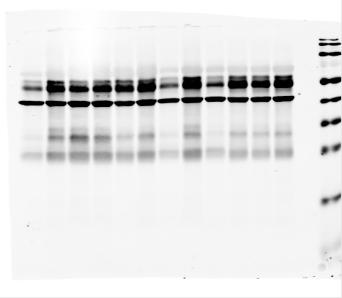

Supplement: Figure 4—source data 2. [file elife-101248-fig4-data2.zip › Figure4-source data2/Figure4g actin.jpg]

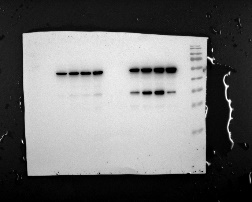

Supplement: Figure 4—source data 2. [file elife-101248-fig4-data2.zip › Figure4-source data2/Figure4g gsdmd.jpg]

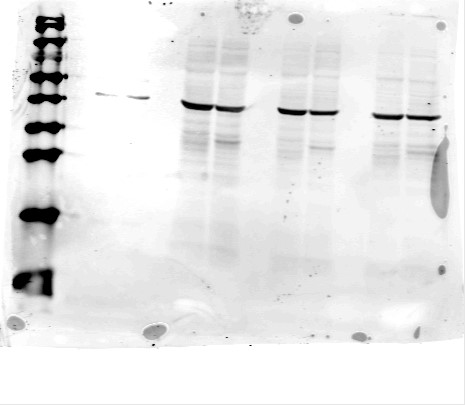

Supplement: Figure 4—source data 2. [file elife-101248-fig4-data2.zip › Figure4-source data2/条敲actin.jpeg]

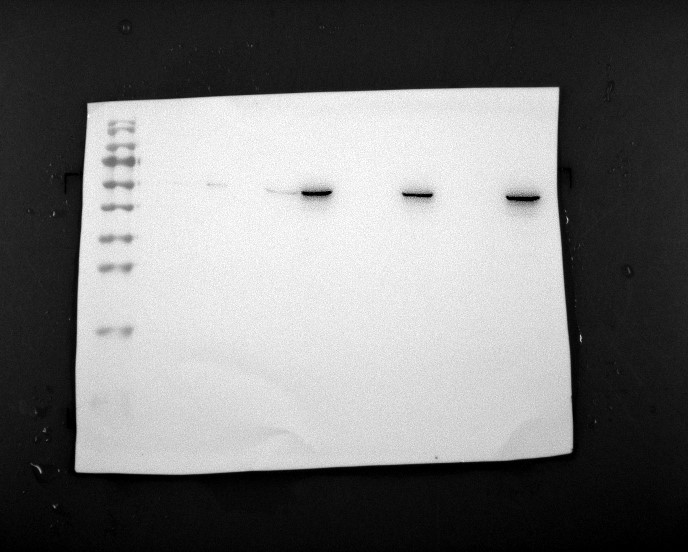

Supplement: Figure 4—source data 2. [file elife-101248-fig4-data2.zip › Figure4-source data2/条敲gsdmd.jpeg]
